# L-form bacteria, chronic diseases and the origins of life

**DOI:** 10.1098/rstb.2015.0494

**Published:** 2016-11-05

**Authors:** Jeff Errington, Katarzyna Mickiewicz, Yoshikazu Kawai, Ling Juan Wu

**Affiliations:** Centre for Bacterial Cell Biology, Institute for Cell and Molecular Biosciences, Newcastle University, Newcastle-upon-Tyne NE24AX, UK

**Keywords:** L-forms, bacterial cell wall, infectious disease, biotechnology, origins of life, *Bacillus subtilis*

## Abstract

The peptidoglycan cell wall is widely conserved across the bacterial domain, suggesting that it appeared early in the evolution of bacteria. It is normally essential but under certain conditions wall-deficient or ‘L-form’ bacteria can be isolated. In *Bacillus subtilis* this normally requires two genetic changes. The first, exemplified by mutations shutting down wall precursor synthesis, works by increasing membrane synthesis. This promotes the unusual form of proliferation used by L-forms, involving a range of relatively disorganized membrane blebbing or vesiculation events. The secondary class of mutations probably work by relieving oxidative stress that L-forms may incur due to their unbalanced metabolism. Repression or inhibition of cell wall precursor synthesis can stimulate the L-form transition in a wide range of bacteria, of both Gram-positive and -negative lineages. L-forms are completely resistant to most antibiotics working specifically on cell wall synthesis, such as penicillins and cephalosporins, consistent with the many reports of their involvement in various chronic diseases. They are potentially important in biotechnology, because lack of a wall can be advantageous in a range of production or strain improvement applications. Finally, L-forms provide an interesting model system for studying early steps in the evolution of cellular life.

This article is part of the themed issue ‘The new bacteriology’.

## 1. The bacterial cell wall

The peptidoglycan (PG) cell wall is one of the defining structures of bacterial cells. The genes encoding the enzymes for its synthesis are conserved and are present in all major bacterial lineages, suggesting that the wall emerged very early in evolution, and it may have been pivotal in enabling the early bacterial radiation [1,2]. The wall is a crucial determinant of bacterial cell shape. It is an elastic structure that confines the cell membrane, counteracting the outward osmotic pressure and enabling the maintenance of turgor in the cell structure [1]. The wall is also crucial for cell division, and many components of the FtsZ-dependent cell division machinery, which is again conserved virtually throughout the bacteria, are concerned with synthesis of cell wall material at the division site [3]. The wall is an important target for antibiotics, such as β-lactams and glycopeptides, and fragments of the wall are recognized by innate immune receptors, helping to trigger powerful immune responses to infection [4,5].

Despite the evident importance of the wall, some groups of bacteria do not possess the genes for wall synthesis. One major group of wall-deficient bacteria is the Tenericutes, including plant and animal symbionts and pathogens, such as *Mycoplasma* and *Phytoplasma*. These organisms are generally highly adapted to life within a eukaryotic host organism. The group almost certainly evolved by reductive evolution from the wall-proficient ancestors of modern Clostridia, losing many genes that are unnecessary for life in the sheltered intracellular environment [6,7].

The main constituent of the cell wall is PG, which is a meshwork made up of long glycan strands cross-linked at frequent intervals by short peptide bridges [8]. The precursor for PG synthesis is a molecule called lipid II, a disaccharide pentapeptide, which is made in the cell cytoplasm by a series of well-characterized enzymes that are found only in the bacteria [9]. Lipid II is flipped to the outside of the membrane [10–12], where it is inserted into the existing PG meshwork to expand the cell wall surface. The glycosyl transferase and transpeptidation reactions needed to attach lipid II to the existing glycan strands are carried out by penicillin-binding proteins, a family of enzymes with their major catalytic domains outside the cell membrane, and are targeted by penicillins and other β-lactam antibiotics [13]. Other antibiotics interfere with wall synthesis by inhibiting various enzymes involved in lipid II synthesis, recycling or insertion into the wall [14]. Finally, various hydrolytic enzymes can dismantle the cell wall, including naturally occurring autolytic enzymes that are required to enlarge the cell surface during growth [15,16], and exogenous enzymes such as lysozyme that are used as defensive agents by the innate immune system [5].

## 2. History of L-form bacteria and possible role in infectious disease

**Figure 1.**
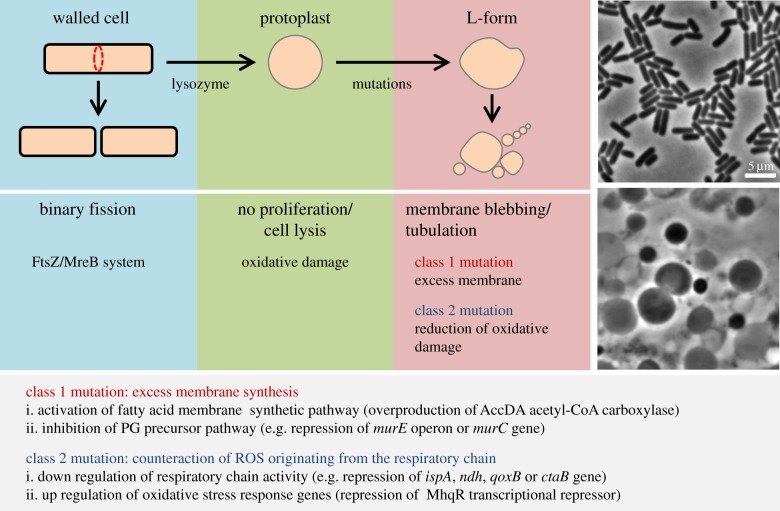
Schematic of the key steps in the walled to L-form transition. Walled cells of rod-shaped bacteria (left) typically grow by elongation of the cell cylinder, governed by the MreB cytoskeletal system, followed by FtsZ-dependent division at mid cell. Lysozyme treatment (for example) (second from left) removes the cell wall, leading to formation of protoplasts, but these are unable to grow due to oxidative damage. Mutations that, in the case of aerobically growing *B. subtilis*, fall into two classes, enable L-form growth (third from left). The right hand panels show phase contrast images of *B. subtilis* in the walled (upper) and L-form (lower) states. Text at bottom of figure lists some of the mutational lesions characteristic of classes 1 and 2.

In the light of the importance of the cell wall, it is surprising that many bacteria are apparently able to switch into a wall-deficient state called the L-form (figure 1). These cells were named in 1935 by Emmy Klieneberger [17]. She was attempting to isolate pleuropneumonia-like organisms (PLOs; now called mycoplasma) from the blood of rats but instead isolated a Gram-negative bacterium called *Streptobacillus moniliformis*. Klieneberger noticed that among the classical rod-shaped bacteria within her culture, pleomorphic organisms were also present, which she assumed to be symbiotic PLOs. Subsequent time-course experiments performed by her colleague, Louis Dienes, revealed that the pleomorphic variants had actually developed from bacilli, and that the bacterium had the ability to switch between the two morphological forms [18].

Klieneberger called the unusual variants L-forms in honour of the Lister Institute in London, where she worked at the time of the discovery. Over the years numerous other names have been ascribed to L-forms, including L-phase bacteria, L-variants, L-organisms and CWD (cell wall-deficient) bacteria [19]. The term L-form is now impossible to define precisely. We presently use it loosely to describe variants of normally walled bacteria that have adapted to grow in the complete absence of cell wall synthesis. As described below, this has important physiological and genetic consequences for the wide range of bacteria that can carry out this switch, including loss of regular shape, osmotic sensitivity, resistance to many wall-targeting antibiotics and ability to tolerate complete deletion of genes involved in PG synthesis and of the FtsZ-based cell-division apparatus [20,21]. Cells treated in various ways to remove the cell wall, sometimes called protoplasts or spheroplasts, can operationally be distinguished from L-forms by their inability (unlike L-forms) to grow and proliferate indefinitely. ‘Stable’ L-forms have picked up mutations that prevent them from reverting to the walled state, whereas ‘unstable’ L-forms can revert, albeit often only at low frequency. Finally, the term L-form has also recently been applied to cells with a partial inhibition of cell wall synthesis (e.g. [22–24]), but it is worth noting that these cells may be physiologically quite distinct from completely wall-deficient L-forms in retaining the requirement for a functional FtsZ-based division machine [22] (see §3).

Following Klieneberger's discovery, L-form-like structures have been observed in samples obtained from humans, animals and plants [19]. However, it has proved challenging to isolate and culture naturally occurring L-forms due to their intrinsically delicate nature. Over the years, researchers realized that the L-form state can be induced experimentally in many bacterial species by treatment of cells with antibiotics, lytic enzymes and/or certain amino acids, which interfere with the bacterial cell wall or its synthesis. The majority of L-forms require osmoprotective conditions for growth, which can be achieved by addition of osmolytes, typically, sucrose or salt, to culture media. L-form growth may also be promoted by other media components, such as magnesium or serum. For unknown reasons, L-forms tend to grow more robustly on solid or semi-solid media.

An important question concerns the pathogenicity of L-forms. Their association with a wide range of infectious diseases has been extensively reviewed [25–28]. The majority of reports focus on persistent or recurrent infections of the urinary, cardiovascular and cerebrospinal systems. However, infections of respiratory, gastrointestinal, integumentary and reproductive systems have also been described. Owing to space constraints, this review focuses on a few specific examples.

The human renal medulla represents a hypertonic physiological environment [29]. Furthermore, in patients suffering from bacterial infections of the bladder and kidney, the osmolarity of urine is often higher than in healthy individuals [30]. It is, therefore, not surprising that many studies have focused on the possibility that L-forms are important in patients with recurrent urinary tract infections and contribute to disease. To isolate L-forms from urine or kidney homogenates researchers historically relied on the ability of L-forms to pass through a 0.45 µm filter, which walled bacterial forms are generally unable to do. Filtered samples were inoculated into media with or without osmoprotection, followed by incubation for prolonged periods. Using this approach, Gutman *et al.* [31] successfully isolated L-forms of *Escherichia coli*, *Klebsiella* spp. and *Enterococcus faecalis* from 11 of 57 patients suffering from chronic bacteriuria or pyelonephritis. Similar results were obtained by various other authors [32–35]. Persistence of *E. facealis* L-forms was tested in a rat model, in which the animals were infected with walled bacterial forms and then treated with penicillin [36,37]. The number of bacterial colonies recovered from the rat after treatment with the antibiotic was higher on osmoprotective media than on media without osmoprotection. 13 weeks after penicillin treatment was withdrawn the only organisms recovered were on osmoprotective media, leading the authors to conclude that L-forms were better able to survive in the rats. Positive results were obtained in a similar model involving rabbits challenged with *Staphylococcus aureus* and then treated with penicillin to induce L-forms *in vivo* [38]. Unfortunately, as reviewed by Clasener [39], other groups using similar methods obtained less convincing results.

Domingue and co-workers have described a series of studies in which L-form-like structures were detected by microscopic methods in the urine and blood of patients suffering from renal diseases and urinary tract infections (reviewed in [26,28]). Using human embryonic kidney fibroblasts (HEK) and axenic animals infected with *E. faecalis*, *Proteus mirabilis* or *E. coli* L-forms to study the ability of L-forms to persist within host tissues, they proposed that small, electron-dense, non-vesiculated forms inside host cells are core elements of bacterial persistence. Consistent with these reports, Wittler *et al.* [40] also detected small granule-like forms in the blood of a patient with subacute endocarditis during treatment with antibiotics. The granules exhibited L-form-like growth in osmoprotective media and later reverted to walled *Corynebacterium*. Almenoff *et al.* [41] were able to isolate CWD *Mycobacterium tuberculosis* from the blood samples of 19 of 20 patients with sarcoidosis, while no growth emerged from the blood of healthy controls. Similar results were obtained for patients with chronic staphylococcal infections [42–44], brucellosis [45] and rheumatic fever caused by group A streptococci [46].

Beaman and co-workers published a series of thorough and elegant reports examining the possible role of *Nocardia* L-forms in disease [47–52]. They showed that in a mouse model of mycetoma *Nocardia caviae* and *N. asteroides* L-forms arose from walled bacteria. The L-forms acted as persisters and were involved in the development of granules characteristic of chronic mycetoma. Interestingly, L-forms were observed only in immunocompetent mice but not in asplenic or athymic mice, suggesting a role for the immune system in the induction of *Nocardia* L-forms *in vivo*. The authors were also able to induce and recover L-forms of *N. asteroides* and *N. caviae* from cultured mouse peritoneal macrophages and lungs, respectively. Moreover, they managed to isolate *N. asteroides* L-forms using osmoprotective media from the cerebrospinal fluid of a patient who later died of brain abscesses. Importantly, no growth was observed on media without osmoprotection, suggesting that, at the time of culture, L-forms were the predominant or only variant of *Nocardia* present in the patient's cerebrospinal fluid. Other studies have looked for an association of CWD bacteria with chronic infections of the central nervous system but with inconclusive results (e.g. [53]).

As the bacterial cell envelope is highly immunogenic, several groups have tried to elucidate whether or not CWD bacteria evoke an immune response and, if so, how this response differs from that triggered by bacteria harbouring a cell wall (reviewed in [19]). In general, these reports show that L-forms are indeed highly immunogenic, but most of the work is difficult to interpret in the light of our modern understanding of immune mechanisms, particularly innate immune responses. Among the more recent papers, Schnell *et al.* [54] observed that *L. monocytogenes* L-forms could be phagocytosed by non-activated murine macrophage-like P388D1 cells and approximately 1% survived intracellularly for up to 72 h post infection. However, activated and bone marrow-derived macrophages cleared the L-forms effectively. The authors reported a strong MyD88-dependent immune response, suggesting an exclusive role for the Toll-like-receptor pathway in recognition of *L. monocytogenes* L-forms by macrophages. In mouse challenge experiments, intraperitoneal inoculation with L-forms resulted in induction of inflammatory cytokines, interleukin-6 and monocyte chemoattractant protein-1. However, the relative magnitude of these responses, compared with walled cells is difficult to estimate, not least because quantifying the viable L-form population is fraught with difficulty (extreme variation in cell size, extent of viable versus non-viable cells, etc.). Moreover, as explained below, our understanding of the relevant physiological state in which to prepare the L-forms and challenge the animals is presently poor.

Nevertheless, from the examples cited in the papers and reviews above it seems plausible that L-form bacteria may be found in humans and could have a role in persistence. Although they are generally thought to be reduced in virulence they could provide a reservoir hidden from the immune system and resistant to treatment with cell-wall-specific antibiotics, from which walled, highly virulent bacteria can emerge.

Despite large amounts of literature published on L-forms, their role in disease remains controversial. Many reports are case studies and lack statistical power, while others could not be repeated. Rigorous controls can be difficult to provide, making it challenging to interpret the results. An additional complication is that many publications were written in languages other than English and are thus not easily accessible to the global scientific community. The majority of publications date back to the pre-molecular era, which means that the methods available to address the central questions of L-form pathogenicity were limited. Reports often relied on simple microscopic observations performed on pathological samples obtained from humans or animals. Owing to their polymorphic nature, it is very easy to confuse L-forms with structures of eukaryotic origin, such as granules, apoptotic bodies or other membrane vesicles, which are particularly abundant in diseased tissues. Non-specific dyes such as acridine orange or DAPI were often used to show that L-form-like structures contained DNA; however, such dyes would stain eukaryotic particles containing DNA equally well. To unambiguously distinguish between structures of bacterial and eukaryotic origin, more specific methods available today could be used, such as fluorescent *in situ* hybridization (FISH) with general probes for bacterial 16s rDNA. Specific probes could then be used to establish the identity of the bacteria.

Reports on L-forms often lacked follow-up experiments due to difficulties in cultivation. L-forms grow much more slowly than their walled counterparts, so walled bacteria present in samples can quickly become dominant. Addition of cell-wall-specific antibiotics or osmoprotective agents to the culture medium can lead to induction of *de novo* L-forms from originally walled bacteria. In such cases, it is impossible to distinguish between L-forms that are present in the original sample and those that were induced in response to the culture conditions. Using filters to separate L-forms from classical bacteria may not always be reliable. Problems with contamination can be significant, especially when long periods of incubation are necessary. Finally, in principle, some L-forms may not grow at all outside the host environment, owing to dependence on factors only available in special intracellular or tissue specific niches. Culture studies need to be combined with careful microscopic observation to demonstrate the presence of L-forms in original samples. Whole genome sequencing and single cell genomics can now be used to demonstrate that the same organism is present in an original sample as was cultured, or even to determine the identity of non-culturable organisms.

Historically, animal and tissue culture studies have not taken into consideration the possibility that incidents of bacterial recurrence could have been due to the presence of other types of persistent cells that have been recognized only recently [55]. Single cell fluorescent time-lapse microscopy could be employed to demonstrate that it is L-forms that survive and revert to classical bacteria in host cells.

Finally, more detailed analysis is needed to determine whether L-forms themselves are pathogenic, or whether they only act as persisters. In the light of their potentially harmless nature and immunomodulatory properties, it could be interesting to explore the potential of L-forms as vaccines against bacterial infection.

Repetition of experiments performed in the 1960s, 1970s and 1980s using new genetic and molecular methods, combined with thorough reports including patient history, *in vivo* and *in vitro* data, as well as large patient studies are needed to unambiguously reveal the significance of L-forms in disease. As described in §3, huge progress has been made in our understanding of the genetics and cell biology of L-forms created in experimental conditions. It remains to be determined whether L-forms that exist in humans follow the same rules.

## 3. Molecular biology of L-forms

Despite the potential importance of L-forms in a wide range of chronic infectious diseases, as described in §2, surprisingly little was known about the molecular biology of L-forms until recently. Siddiqui *et al.* [56] reported that partial sequencing of a classically derived *E. coli* L-form [57] revealed mutations in several wall and division associated genes. Unfortunately, this strain had been propagated in the L-form state for several decades, so it was not clear whether the mutations were associated with the initial L-form switch or selected secondarily during propagation. Our laboratory took the approach of examining the genetic changes associated with the initial switch from walled to L-form state, using *Bacillus subtilis* as a model system.

Almost all bacterial cells in their normal walled state divide by binary fission via a complex protein-based FtsZ division machinery [3]. Instead, L-forms proliferate by a range of poorly regulated shape perturbations, including blebbing, tubulation and vesiculation [20,58–60] (figure 1). Remarkably, the work on *B. subtilis* has revealed that L-forms do not require the normally essential FtsZ division machinery for their proliferation [20]. FtsZ independent cell growth was then also shown for L-forms of *E. coli* and other bacteria (see below) [21]. Attempts to identify cytoskeletal proteins or motor proteins that might actively drive cell division events in L-forms were negative, suggesting the existence of a simpler mechanism. The first mutations found that specifically interfered with L-form growth turned out apparently to work by reducing membrane fluidity. This resulted in a failure in separation or ‘scission’ of progeny cells [61] and pointed to the likely importance of the membrane dynamics in L-form proliferation.

The molecular basis underlying L-form proliferation became apparent as a result of the recent identification of genetic requirements for their production and proliferation in *B. subtilis* [20,62–64] (figure 1). The key experiments were done by taking wild-type cells and stripping the PG wall by treatment with lysozyme, in the presence of sucrose to prevent cell lysis. For reasons that are only just being clarified (see below), the resultant protoplasts do not undergo significant growth, but variants able to grow in this state, i.e. L-forms, could be selected and characterized. The results of an extensive series of genetic experiments revealed that in *B. subtilis* a combination of two kinds of mutations is required to enable L-form growth under laboratory conditions [63]. Class 1 mutations generate excess amounts of cell membrane, either by directly activating the fatty acid membrane synthetic pathway, or indirectly, by shutting down PG precursor pathway, which works via an as yet uncharacterized mechanism [63]. The L-form proliferation promoted by class 1 mutations could be blocked by a minor reduction in membrane synthesis (that would have no effect on cell growth in the parental walled cells), confirming the importance of excess membrane synthesis for L-form proliferation [63]. Strikingly, it was also shown that artificially increasing cell surface area by the conversion of elongated rods to spherical protoplasts was sufficient to induce L-form-like shape perturbations and scission in wild-type cells. Together, the results led to a simple model in which L-form proliferation is brought about by an imbalance between cell membrane and volume growth, which drives cell shape deformations leading to scission [63]. This work also highlighted the possibility that L-form proliferation is driven by simple biophysical processes involving membrane dynamics, and thus L-forms may represent a primitive mode of cell proliferation that was used early in the evolution of cellular life, before the invention of the PG wall [2,20,60] (see §5).

The class 2 mutations required for L-form proliferation were found to work either by downregulating respiratory chain activity, or upregulating oxidative stress response genes [64]. Thus, the class 2 mutations seem to support L-form growth by counteracting an increase in the cellular levels of reactive oxygen species (ROS) originating from the respiratory chain. We presume that this is due to a metabolic imbalance in the wall-deficient cells, and that oxidative damage is a serious impediment to L-form proliferation [64]. Indeed, several antioxidant systems, such as catalase and superoxide dismutase, were essential for L-form growth under aerobic conditions, but not in the parental walled cells. Consistent with these ideas, the class 2 mutations were no longer required for L-form growth when oxygen was depleted. *Escherichia coli* L-form growth was also stimulated under anaerobic conditions or by adding a ROS scavenger, suggesting that oxidative damage might be an important impediment to L-form growth in a wide range of bacteria [64].

The molecular basis of L-form proliferation established by *B. subtilis* studies mentioned above seems to be conserved across a wide range of bacteria, based on the following observations: (i) in several bacterial species, including both Gram-positives (the Firmicute *S. aureus* and the Actinobacterium *Corynebacterium glutamicum*), and the Gram-negative γ-proteobacterium *E. coli*, the L-form transition is efficiently promoted by repression of PG precursor synthesis; (ii) crucially, the cells do not require the normally essential cell division machinery for proliferation; and (iii) a minor reduction in fatty acid synthesis blocks L-form growth [21]. The regulation of membrane synthesis seems, therefore, to have a pivotal role in the L-form proliferation of diverse bacteria, and thus it is likely that an increased ratio of surface area to volume synthesis is a common mechanism supporting the proliferation of L-form bacteria. Nevertheless, it remains possible that the mechanism of L-form transition is more complex and less well conserved than is apparent from the above results. Work from the Loessner lab with *L. monocytogenes* and *E. faecalis* has highlighted a slightly different mode of proliferation of L-forms, in which large L-forms acquire complex internal vesicles which are released as progeny cells upon lysis of the ‘mother’ L-form [59,60]. These experiments were done within semi-solid agar, so it could be that the internal vesiculation is driven by the constraining agar milieu. Briers *et al.* [60] also reported that a ‘stable’ *L. monocytogenes* L-form did not carry ‘predisposing’ mutations. However, the strain did have mutations affecting glycolysis and one pathway leading to bactoprenol synthesis. Given that it is unable to revert to the walled state it seems likely that it contains mutations preventing wall synthesis, which could conceivably promote L-form growth in a similar manner to the pathways described above. Further work is needed to establish whether the different findings are due to trivial differences in experimental methods or more fundamental variations between organisms.

The ability to rebuild a PG cell wall *de novo* is also a common property of L-forms [19,21,23,24,65]. Reversion to the walled form is presumably important for pathogenicity in patients with persistent or recurrent infections. Although the molecular mechanisms underlying cell wall regeneration remain poorly understood, L-forms could provide a vehicle for investigating *de novo* cell wall synthesis, and also cell morphogenesis.

Identification of the universally essential genes or biological pathways in L-forms across the bacterial domain could contribute to our understanding of the principles underpinning the L-form state. Mutant screens, using deletion or transposon libraries, to identify genes involved in formation or growth of L-form colony have been attempted for *E. coli* [23,66], *S. aureus* [67] and *B. subtilis* [61], and the results have identified several genes specifically required for L-form growth. Although the mode of L-form proliferation seems to be driven by a common mechanism, as described above, no clear pattern of conserved genes required for L-form proliferation has as yet emerged. It might be that the processes of L-form formation vary between bacterial species, or the differences may stem from the methods used to generate and propagate L-forms in the various different laboratories.

## 4. Potential biotechnology uses of L-forms

Based on our improved understanding of the molecular cell biology of L-forms, it is timely to consider whether their unusual properties might have biotechnological applications. Protoplast fusion and protoplast transformation are longstanding methods that have been used to generate recombinant progeny cells [68,69]. A key step with both methods lies in the regeneration of walled cells, which is often inefficient. The ability of L-forms to grow indefinitely without regeneration may be advantageous in recovering certain kinds of recombinants. It is plausible that L-forms could be particularly effective for extreme forms of recombinant recovery, such as whole genome ‘rebooting’ [70].

A second area in which L-forms could be exploited relates to situations in which the wall is an impediment to product accumulation. As mentioned in §2, fragments of cell wall PG can trigger powerful innate immune responses, so for various biomedical applications it is crucial that PG is quantitatively removed during downstream processing (e.g. for therapeutic proteins or peptides). For these applications, the cells could be grown in the L-form state in the complete absence of PG synthesis. Alternatively, to achieve more robust biomass accumulation, cells could initially be grown in the walled state and then switched to L-forms later in production by turning on or off the appropriate genes. The cell wall is also a potential barrier to the export of secreted proteins. Indeed, the wall can strongly influence the folding and stability of secreted proteins, particularly those of heterologous origin [71]. Thus, turning on protein secretion in L-form cells might lead to substantial improvements in product yield.

A third class of application for L-forms relies on their characteristic production of excess membrane. Membrane surface area is presumably a crucial limiting factor in the accumulation of hydrophobic small molecules (e.g. fatty acids and lipids) or membrane spanning proteins. Therefore, it should be possible to improve the yield of at least some hydrophobic commercial products by producing them in L-forms. Protein secretion might also be facilitated through this effect in respect of the increased surface area over which secretion can take place.

In general, the diversion of metabolic energy away from the synthesis of PG and potentially other cell wall molecules could enhance the yield of a wide range of products, especially in Gram-positive bacteria in which the wall represents a substantial proportion of total cell mass. However, against this advantage should be weighed the disadvantages of the relative fragility and lowered overall growth rate of L-forms. Ultimately, it seems likely that applications for L-forms will be limited to higher added value products, rather than bulk commodity items.

Some of these areas of potential application for L-forms have been explored previously, particularly by Gumpert & Hoischen [72]. Our ability to control the key genetic changes needed for the switch to L-form growth, together with our improved understanding of their physiology, should facilitate the development of commercial applications in the future.

## 5. L-forms and the origins of life

Current thinking on the evolutionary pathway leading to the emergence of life identifies a series of key steps (reviewed in [73]). The first lies in emergence of the key building blocks of life, such as amino acids and nucleotides. Then, it is envisaged that an RNA-based world emerged, in which RNA acted as both information store and catalyst. These dual properties would have provided a mechanism for natural selection or Darwinian evolution to come into effect, leading to the development of ever more sophisticated and efficient RNA-based proto-organisms. This process, however, would seem to have required the existence of localized containment, so that improved RNA molecules could benefit from the products of their action. It is assumed that this step involved semi-permeable vesicular structures, presumably composed of fatty acids or lipids. Crucially, these vesicles would need to be capable of replication with limited loss of material during the transition from parental to progeny vesicles. As described in §3, the L-form mode of proliferation appears remarkably simple in that it requires only an increased rate of membrane synthesis (compared with the rod-shaped parental cells), and not to require any protein-based mechanical apparatus, such as the FtsZ-based division machinery [61,63]. As it appears that many diverse bacteria can accomplish the switch from walled to non-walled state in a similarly simple manner, it is plausible that the L-form mode of proliferation may be a good model for the proliferation of primitive cells before the invention of the PG cell wall. *In vitro* and theoretical studies support the idea of simple vesicle replication being driven by an increase in surface area to volume ratio [74,75], so there seems to be a convergence of top down and bottom up approaches to this origins of life problem. Phylogenetic analyses of the bacterial lineage suggest that the PG cell wall is a very ancient structure. Given that primordial L-form-like cells would have been fragile and sensitive to changes in osmolarity and desiccation, it is conceivable that invention of the cell wall was a pivotal moment in the evolution of cellular life, enabling the first true bacteria to spread out from well protected ‘nursery’ environments and colonise the planet. As pointed out by an anonymous reviewer, this leads to a testable prediction that the central enzymes in membrane biogenesis should have evolved earlier than those of PG, which we plan to investigate.

At the moment some of the other key questions posed by this thinking lie around the coupling between vesicle replication and chromosome replication and segregation. Based on theoretical and *in vitro* experiments (e.g. [76,77]), it is possible that replicated and segregated chromosomes have a role in directing the formation of vesicular blebs and tubules. However, further experiments are needed to verify these findings and to test directly the effect of perturbations in replication and segregation on L-form proliferation.

## References

[1] Koch AL 2006 The exocytoskeleton. J. Mol. Microbiol. Biotechnol. 11, 115–125. (10.1159/000094048) 16983189

[2] Errington J 2013 L-form bacteria, cell walls and the origins of life. Open Biol. 3, 120143 (10.1098/rsob.120143) 23303308 PMC3603455

[3] Adams DW, Errington J 2009 Bacterial cell division: assembly, maintenance and disassembly of the Z ring. Nat. Rev. Microbiol. 7, 642–653. (10.1038/nrmicro2198) 19680248

[4] Akira S, Uematsu S, Takeuchi O 2006 Pathogen recognition and innate immunity. Cell 124, 783–801. (10.1016/j.cell.2006.02.015) 16497588

[5] Sukhithasri V, Nisha N, Biswas L, Anil Kumar V, Biswas R 2013 Innate immune recognition of microbial cell wall components and microbial strategies to evade such recognitions. Microbiol. Res. 168, 396–406. (10.1016/j.micres.2013.02.005) 23578963

[6] Woese CR, Maniloff J, Zablen LB 1980 Phylogenetic analysis of the mycoplasmas. Proc. Natl Acad. Sci. USA 77, 494–498. (10.1073/pnas.77.1.494) 6928642 PMC348298

[7] Gundersen DE, Lee IM, Rehner SA, Davis RE, Kingsbury DT 1994 Phylogeny of mycoplasmalike organisms (phytoplasmas): a basis for their classification. J. Bacteriol. 176, 5244–5254. 8071198 10.1128/jb.176.17.5244-5254.1994 PMC196707

[8] Höltje JV 1998 Growth of the stress-bearing and shape-maintaining murein sacculus of *Escherichia coli*. Microbiol. Mol. Biol. Rev. 62, 181–203. 9529891 10.1128/mmbr.62.1.181-203.1998 PMC98910

[9] Barreteau H, Kovac A, Boniface A, Sova M, Gobec S, Blanot D 2008 Cytoplasmic steps of peptidoglycan biosynthesis. FEMS Microbiol. Rev. 32, 168–207. (10.1111/j.1574-6976.2008.00104.x) 18266853

[10] Mohammadi T et al. 2011 Identification of FtsW as a transporter of lipid-linked cell wall precursors across the membrane. EMBO J. 30, 1425–1432. (10.1038/emboj.2011.61) 21386816 PMC3102273

[11] Sham LT, Barendt SM, Kopecky KE, Winkler ME 2011 Essential PcsB putative peptidoglycan hydrolase interacts with the essential FtsXSpn cell division protein in *Streptococcus pneumoniae* D39. Proc. Natl Acad. Sci. USA 108, E1061–E1069. (10.1073/pnas.1108323108) 22006325 PMC3215045

[12] Meeske AJ, Sham LT, Kimsey H, Koo BM, Gross CA, Bernhardt TG, Rudner DZ 2015 MurJ and a novel lipid II flippase are required for cell wall biogenesis in *Bacillus subtilis*. Proc. Natl Acad. Sci. USA 112, 6437–6442. (10.1073/pnas.1504967112) 25918422 PMC4443310

[13] Lovering AL, Safadi SS, Strynadka NC 2012 Structural perspective of peptidoglycan biosynthesis and assembly. Annu. Rev. Biochem. 81, 451–478. (10.1146/annurev-biochem-061809-112742) 22663080

[14] Silver LL 2013 Viable screening targets related to the bacterial cell wall. Ann. NY Acad. Sci. 1277, 29–53. (10.1111/nyas.12006) 23278681

[15] Vollmer W, Joris B, Charlier P, Foster S 2008 Bacterial peptidoglycan (murein) hydrolases. FEMS Microbiol. Rev. 32, 259–286. (10.1111/j.1574-6976.2007.00099.x) 18266855

[16] van Heijenoort J 2011 Peptidoglycan hydrolases of *Escherichia coli*. Microbiol. Mol. Biol. Rev. 75, 636–663. (10.1128/MMBR.00022-11) 22126997 PMC3232740

[17] Klieneberger E 1935 The natural occurrence of pleuropneumonia-like organisms in apparent symbiosis with *Streptobacillus moniliformis* and other bacteria. J. Pathol. Bacteriol. 40, 93–105. (10.1002/path.1700400108)

[18] Dienes L 1939 L Organisms of Klieneberger and *Streptobacillus moniliformis*. J. Infect. Dis. 65, 24–42. (10.1093/infdis/65.1.24)

[19] Allan EJ, Hoischen C, Gumpert J 2009 Bacterial L-forms. Adv. Appl. Microbiol. 68, 1–39. (10.1016/S0065-2164(09)01201-5) 19426852

[20] Leaver M, Dominguez-Cuevas P, Coxhead JM, Daniel RA, Errington J 2009 Life without a wall or division machine in *Bacillus subtilis*. Nature 457, 849–853. (10.1038/nature07742) 19212404

[21] Mercier R, Kawai Y, Errington J 2014 General principles for the formation and proliferation of a wall-free (L-form) state in bacteria. eLife 3, 642 (10.7554/eLife.04629) PMC4244569 25358088

[22] Joseleau-Petit D, Liebart JC, Ayala JA, D'Ari R 2007 Unstable *Escherichia coli* L forms revisited: growth requires peptidoglycan synthesis. J. Bacteriol. 189, 6512–6520. (10.1128/JB.00273-07) 17586646 PMC2045188

[23] Cambré A et al. 2015 Metabolite profiling and peptidoglycan analysis of transient cell wall-deficient bacteria in a new *Escherichia coli* model system. Environ. Microbiol. 17, 1586–1599. (10.1111/1462-2920.12594) 25142185

[24] Billings G, Ouzounov N, Ursell T, Desmarais SM, Shaevitz J, Gitai Z, Huang KC 2014 *De novo* morphogenesis in L-forms via geometric control of cell growth. Mol. Microbiol. 93, 883–896. (10.1111/mmi.12703) 24995493 PMC4459576

[25] Clasener H 1972 Pathogenicity of the L-phase of bacteria. Annu. Rev. Microbiol. 26, 55–84. (10.1146/annurev.mi.26.100172.000415) 4562818

[26] Domingue GJ Sr, Woody HB 1997 Bacterial persistence and expression of disease. Clin. Microbiol. Rev. 10, 320–344. 9105757 10.1128/cmr.10.2.320 PMC172922

[27] Onwuamaegbu ME, Belcher RA, Soare C 2005 Cell wall-deficient bacteria as a cause of infections: a review of the clinical significance. J. Int. Med. Res. 33, 1–20. (10.1177/147323000503300101) 15651712

[28] Domingue GJ 2010 Demystifying pleomorphic forms in persistence and expression of disease: are they bacteria, and is peptidoglycan the solution? Discov. Med. 10, 234–246. 20875345

[29] Kwon MS, Lim SW, Kwon HM 2009 Hypertonic stress in the kidney: a necessary evil. Physiology (Bethesda, MD) 24, 186–191. (10.1152/physiol.00005.2009) 19509128

[30] Sobel JD 1985 New aspects of pathogenesis of lower urinary tract infections. Urology 26(Suppl. 5), 11–16. 3904135

[31] Gutman LT, Turck M, Petersdorf RG, Wedgwood RJ 1965 Significance of bacterial variants in urine of patients with chronic bacteriuria. J. Clin. Invest. 44, 1945–1952. (10.1172/JCI105300) 4954866 PMC289696

[32] Domingue GJ, Schlegel JU 1970 The possible role of microbial L-forms in pyelonephritis. J. Urol. 104, 790–798. 5499826 10.1016/s0022-5347(17)61838-x

[33] Braude AI, Siemienski J, Jacobs I 1961 Protoplast formation in human urine. Trans. Assoc. Am. Phys. 74, 234–245. 13872570

[34] Kalmanson GM, Hubert EG, Guze LB 1969 Production and therapy of *Proteus mirabilis* pyelonephritis in mice undergoing chronic diuresis. Antimicrob. Agents Chemother. (Bethesda) 9, 458–462. 5396524 10.1128/AAC.9.3.458

[35] Coleman JC, Little PJ 1967 Bacterial variants in the human kidney. J. Pathol. Bacteriol. 94, 213–215. (10.1002/path.1700940130) 4964054

[36] Guze LB, Kalmanson GM 1964 Persistence of bacteria in ‘protoplast’ form after apparent cure of pyelonephritis in rats. Science 143, 1340–1341. (10.1126/science.143.3612.1340) 14113625

[37] Guze LB, Kalmanson GM 1964 Action of erythromycin on ‘protoplasts’ *in vivo*. Science 146, 1299–1300. (10.1126/science.146.3649.1299) 14207454

[38] Young RM, Dahlquist EH 1967 Pathogenicity of L forms of *Staphylococcus aureus*. Am. J. Clin. Pathol. 48, 466–473. (10.1093/ajcp/48.5.466) 6069925

[39] Clasener HA, Ensering HL 1970 Search for the L-phase of *Streptococcus faecalis* in kidneys of rats experimentally infected with the bacterial phase and treated with penicillin. Ann. NY Acad. Sci. 174, 880–895. (10.1111/j.1749-6632.1970.tb45608.x) 4994780

[40] Wittler RG, Malizia WF, Kramer PE, Tuckett JD, Pritchard HN, Baker HJ 1960 Isolation of a *Corynebacterium* and its transitional forms from a case of subacute bacterial endocarditis treated with antibiotics. J. Gen. Microbiol. 23, 315–333. (10.1099/00221287-23-2-315) 13786116

[41] Almenoff PL, Johnson A, Lesser M, Mattman LH 1996 Growth of acid fast L forms from the blood of patients with sarcoidosis. Thorax 51, 530–533. (10.1136/thx.51.5.530) 8711683 PMC473601

[42] Lechner S, Lewis K, Bertram R 2012 *Staphylococcus aureus* persisters tolerant to bactericidal antibiotics. J. Mol. Microbiol. Biotechnol. 22, 235–244. (10.1159/000342449) 22986269 PMC3518770

[43] Rosner R 1968 Isolation of protoplasts of *Staphylococcus aureus* from a case of recurrent acute osteomyelitis. Tech. Bull. Regist. Med. Technol. 38, 205–210. 5678596

[44] Godzeski CW, Brier G, Griffith RS, Black HR 1965 Association of bacterial L-phase organisms in chronic infections. Nature 205, 1340 (10.1038/2051340a0) 14311978

[45] Nelson EL, Pickett MJ 1951 The recovery of L forms of *Brucella* and their relation to *Brucella* phage. J. Infect .Dis. 89, 226–232. (10.1093/infdis/89.3.226) 14888947

[46] Timakov VD, Kagan GYa 1962 L forms of haemolytic streptococcus and their detection in the blood of patients with rheumatic fever and bacterial endocarditis. J. Reumatisma 2, 3–8.

[47] Beaman BL, Shankel DM 1969 Ultrastructure of *Nocardia* cell growth and development on defined and complex agar media. J. Bacteriol. 99, 876–884. 4905542 10.1128/jb.99.3.876-884.1969 PMC250106

[48] Beaman BL 1973 An ultrastructural analysis of *Nocardia* during experimental infections in mice. Infect. Immun. 8, 828–840. 4584055 10.1128/iai.8.5.828-840.1973 PMC422934

[49] Beaman BL, Burnside J, Edwards B, Causey W 1976 Nocardial infections in the United States, 1972–1974. J. Infect. Dis. 134, 286–289. (10.1093/infdis/134.3.286) 789786

[50] Beaman BL, Bourgeois AL, Moring SE 1981 Cell wall modification resulting from *in vitro* induction of L-phase variants of *Nocardia asteroides*. J. Bacteriol. 148, 600–609. 7028720 10.1128/jb.148.2.600-609.1981 PMC216245

[51] Beaman BL 1980 Induction of L-phase variants of *Nocardia caviae* within intact murine lungs. Infect. Immun. 29, 244–251. 7399704 10.1128/iai.29.1.244-251.1980 PMC551102

[52] Beaman BL, Smathers M 1976 Interaction of *Nocardia asteroides* with cultured rabbit alveolar macrophages. Infect. Immun. 13, 1126–1131. 776829 10.1128/iai.13.4.1126-1131.1976 PMC420728

[53] Kenny JF 1973 Bacterial variants in central nervous system infections in infants and children. J. Pediatr. 83, 531–542. (10.1016/S0022-3476(73)80211-2) 4147126

[54] Schnell B, Staubli T, Harris NL, Rogler G, Kopf M, Loessner MJ, Schuppler M 2014 Cell-wall deficient *L. monocytogenes* L-forms feature abrogated pathogenicity. Front. Cell. Infect. Microbiol. 4, 60 (10.3389/fcimb.2014.00060) 24904838 PMC4033035

[55] Kester JC, Fortune SM 2014 Persisters and beyond: mechanisms of phenotypic drug resistance and drug tolerance in bacteria. Crit. Rev. Biochem. Mol. Biol. 49, 91–101. (10.3109/10409238.2013.869543) 24328927

[56] Siddiqui RA, Hoischen C, Holst O, Heinze I, Schlott B, Gumpert J, Diekmann S, Grosse F, Platzer M 2006 The analysis of cell division and cell wall synthesis genes reveals mutationally inactivated *ftsQ* and *mraY* in a protoplast-type L-form of *Escherichia coli*. FEMS Microbiol. Lett. 258, 305–311. (10.1111/j.1574-6968.2006.00237.x) 16640589

[57] Schuhmann E, Taubeneck U 1969 Stabil L-forms of several *Escherichia coli* strains. Z. Allg. Mikrobiol. 9, 297–313. (10.1002/jobm.3630090407) 4903940

[58] Kandler G, Kandler O 1954 Studies on morphology and multiplication of pleuropneumonia-like organisms and on bacterial L-phase, I. Light microscopy. Arch. Mikrobiol. 21, 178–201. (10.1007/BF01816378) 14350641

[59] Dell'Era S, Buchrieser C, Couve E, Schnell B, Briers Y, Schuppler M, Loessner MJ 2009 *Listeria monocytogenes* L-forms respond to cell wall deficiency by modifying gene expression and the mode of division. Mol. Microbiol. 73, 306–322. (10.1111/j.1365-2958.2009.06774.x) 19555455

[60] Briers Y, Walde P, Schuppler M, Loessner MJ 2012 How did bacterial ancestors reproduce? Lessons from L-form cells and giant lipid vesicles: multiplication similarities between lipid vesicles and L-form bacteria. Bioessays 34, 1078–1084. (10.1002/bies.201200080) 23108858

[61] Mercier R, Domínguez-Cuevas P, Errington J 2012 Crucial role for membrane fluidity in proliferation of primitive cells. Cell Rep. 1, 417–423. (10.1016/j.celrep.2012.03.008) 22832271

[62] Domínguez-Cuevas P, Mercier R, Leaver M, Kawai Y, Errington J 2012 The rod to L-form transition of *Bacillus subtilis* is limited by a requirement for the protoplast to escape from the cell wall sacculus. Mol. Microbiol. 83, 52–66. (10.1111/j.1365-2958.2011.07920.x) 22122227

[63] Mercier R, Kawai Y, Errington J 2013 Excess membrane synthesis drives a primitive mode of cell proliferation. Cell 152, 997–1007. (10.1016/j.cell.2013.01.043) 23452849

[64] Kawai Y, Mercier R, Wu LJ, Dominguez-Cuevas P, Oshima T, Errington J 2015 Cell growth of wall-free L-form bacteria is limited by oxidative damage. Curr. Biol. 25, 1613–1618. (10.1016/j.cub.2015.04.031) 26051891 PMC4510147

[65] Kawai Y, Mercier R, Errington J 2014 Bacterial cell morphogenesis does not require a preexisting template structure. Curr. Biol. 24, 863–867. (10.1016/j.cub.2014.02.053) 24704074 PMC3989771

[66] Glover WA, Yang Y, Zhang Y 2009 Insights into the molecular basis of L-form formation and survival in *Escherichia coli*. PLoS ONE 4, e7316 (10.1371/journal.pone.0007316) 19806199 PMC2752164

[67] Han J, Shi W, Xu X, Wang S, Zhang S, He L, Sun X, Zhang Y 2015 Conditions and mutations affecting *Staphylococcus aureus* L-form formation. Microbiology 161, 57–66. (10.1099/mic.0.082354-0) 25361600

[68] Baltz RH 2001 Genetic methods and strategies for secondary metabolite yield improvement in actinomycetes. Antonie Van Leeuwenhoek 79, 251–259. (10.1023/A:1012020918624) 11816967

[69] Chang S, Cohen SN 1979 High frequency transformation of *Bacillus subtilis* protoplasts by plasmid DNA. Mol. Gen. Genet. 168, 111–115. (10.1007/BF00267940) 107388

[70] Gibson DG et al. 2010 Creation of a bacterial cell controlled by a chemically synthesized genome. Science 329, 52–56. (10.1126/science.1190719) 20488990

[71] Harwood CR, Cranenburgh R 2008 *Bacillus* protein secretion: an unfolding story. Trends Microbiol. 16, 73–79. (10.1016/j.tim.2007.12.001) 18182292

[72] Gumpert J, Hoischen C 1998 Use of cell wall-less bacteria (L-forms) for efficient expression and secretion of heterologous gene products. Curr. Opin. Biotechnol. 9, 506–509. (10.1016/S0958-1669(98)80037-2) 9821280

[73] Lilley DM, Sutherland J 2011 The chemical origins of life and its early evolution: an introduction. Phil. Trans. R. Soc. B 366, 2853–2856. (10.1098/rstb.2011.0133) 21930575 PMC3158915

[74] Peterlin P, Arrigler V, Kogej K, Svetina S, Walde P 2009 Growth and shape transformations of giant phospholipid vesicles upon interaction with an aqueous oleic acid suspension. Chem. Phys. Lipids 159, 67–76. (10.1016/j.chemphyslip.2009.03.005) 19477312

[75] Svetina S 2009 Vesicle budding and the origin of cellular life. Chemphyschem 10, 2769–2776. (10.1002/cphc.200900577) 19774545

[76] Terasawa H, Nishimura K, Suzuki H, Matsuura T, Yomo T 2012 Coupling of the fusion and budding of giant phospholipid vesicles containing macromolecules. Proc. Natl Acad. Sci. USA 109, 5942–5947. (10.1073/pnas.1120327109) 22474340 PMC3340996

[77] Yu Y, Granick S 2009 Pearling of lipid vesicles induced by nanoparticles. J. Am. Chem. Soc. 131, 14 158–14 159. (10.1021/ja905900h) 19775107

